# COVID-19 Versus Influenza Hospitalizations in Late 2025 in Poland: A Multicenter Retrospective Comparison

**DOI:** 10.3390/vaccines14030210

**Published:** 2026-02-26

**Authors:** Piotr Rzymski, Dominik Sznajder, Małgorzata Wajdowicz, Anna Moniuszko-Malinowska, Monika Pazgan-Simon, Paweł Skwara, Justyna Hlebowicz, Katarzyna Sikorska, Maciej Piasecki, Robert Flisiak, Dorota Zarębska-Michaluk

**Affiliations:** 1 Department of Environmental Medicine, Poznan University of Medical Sciences, 60-806 Poznań, Poland; rzymskipiotr@ump.edu.pl; 2 Department of Infectious and Tropical Diseases and Acquired Immunodeficiency, Pomeranian Medical University, 70-111 Szczecin, Poland; dominixx02@gmail.com; 3 Medical Center in Łańcut, Clinical Department of Infectious Diseases and Hepatologic Unit, University of Rzeszów, 35-959 Rzeszów, Poland; mwajdowicz@gmail.com; 4 Department of Infectious Diseases and Neuroinfection, Medical University of Bialystok, 15-540 Białystok, Poland; anna.moniuszko-malinowska@umb.edu.pl; 5 Department of Infectious Diseases and Hepatology, Wrocław Medical University, 50-370 Wrocław, Poland; monika.pazgan.simon@gmail.com; 6 Department of Infectious and Tropical Diseases, Jagiellonian University Medical College, 330-688 Kraków, Poland; pawel.skwara@uj.edu.pl; 7 Department of Infectious Diseases and Hepatology, Medical University of Bialystok, 15-540 Białystok, Poland; hs.justyna@gmail.com; 8 Division of Tropical and Parasitic Diseases, Institute of Maritime and Tropical Medicine, Medical University of Gdansk, 80-210 Gdynia, Poland; katarzyna.sikorska@gumed.edu.pl; 9 Department of Infectious Diseases and Hepatology, Medical University of Silesia, 41-500 Katowice, Poland; maciejp67@gmail.com; 10 Department of Infectious Diseases and Allergology, Jan Kochanowski University, 25-317 Kielce, Poland; dorota1010@tlen.pl

**Keywords:** respiratory viral infections, hospitalizations, COVID-19, influenza, viral epidemiology

## Abstract

**Background/Objectives**: COVID-19 and influenza remain major causes of seasonal hospitalizations, with increasing overlap toward the end of the calendar year. Comparative data from this late-year period remain limited, especially for the Central European region. **Methods**: We retrospectively analyzed adult patients hospitalized with COVID-19 or influenza between September and December 2025 in nine infectious disease units in Poland. Demographic, clinical, and laboratory characteristics were compared, and independent predictors of in-hospital mortality were identified using multivariable logistic regression. **Results**: A total of 227 patients with COVID-19 and 87 with influenza were included. COVID-19 hospitalizations predominated in September–October, whereas influenza admissions increased sharply in December. COVID-19 patients were older and more frequently had cancer. Length of hospitalization, baseline oxygen saturation, inflammatory markers, and pneumonia rates were similar between groups. In-hospital mortality was significantly higher among COVID-19 patients (14% vs. 6%). Among patients who died from influenza, none received the latest seasonal vaccine; among patients who died from COVID-19, 84.4% did not receive an updated COVID-19 vaccine. After adjustment, COVID-19 was independently associated with a fourfold increased risk of death, along with hypoxemia and pneumonia. **Conclusions**: During the last quadrimester of 2025, COVID-19 caused more hospitalizations and higher in-hospital mortality than influenza in Poland. Though other studies have evidenced that COVID-19 severity decreased, it remains to cause a substantial healthcare burden. Our findings also suggest earlier COVID-19 vaccination in Poland (initiated preferentially before September) and enhanced healthcare preparedness during late-year periods of viral co-circulation.

## 1. Introduction

Seasonal respiratory viral infections remain a major cause of morbidity and mortality, placing a substantial burden on healthcare systems worldwide [1,2]. Influenza has traditionally been regarded as the dominant seasonal viral threat, with predictable winter peaks and well-established vaccination strategies. However, since the emergence of SARS-CoV-2, COVID-19 has become an additional and persistent contributor to hospital admissions, severe disease, and death, particularly among older adults and individuals with chronic comorbidities [3,4]. Beyond its direct impact, the COVID-19 pandemic appears to have altered the broader epidemiology of respiratory viruses, influencing patterns of circulation, seasonality, and healthcare utilization [5,6,7,8]. As population immunity, vaccination behaviors, and viral interactions continue to evolve, the post-pandemic period is characterized by a dynamic, less predictable landscape of respiratory infections [9,10,11,12].

While influenza activity in Europe typically increases substantially at the beginning of the new year [13], recent data suggest the possibility of earlier seasonal circulation. For example, in 2024, influenza hospitalizations in Poland rose sharply in the last months of the year, with influenza-related mortality in December 2024 nearly doubling the total annual admissions observed in 2023 [14]. These findings underscore the vulnerability of healthcare systems at the end of the calendar year and challenge the assumption that the major influenza burden, manifested by hospitalizations, occurs exclusively after January. In parallel, COVID-19 continues to cause significant disease burden outside of classical epidemic seasonality, with meaningful infections observed year-round and peaks in summer and winter [15]. However, although comparative studies of COVID-19 and influenza hospitalizations exist [16,17,18], data directly contrasting both infections within the same clinical setting during the late-year period preceding the typical influenza peak remain limited.

Therefore, the present study aimed to compare the clinical characteristics, hospitalization burden, and mortality of patients admitted with COVID-19 and influenza during the last quadrimester of 2025 in Poland, a period of increasing viral co-circulation and increased strain on the healthcare system.

## 2. Materials and Methods

Data on demographic characteristics (age, sex), comorbidities, clinical parameters (oxygen saturation, serum C-reactive protein (CRP) levels, development of pneumonia), and vaccination status of adult patients hospitalized with COVID-19 and influenza in September–December 2025 were collected retrospectively from nine infectious disease units across Poland (two in Białystok, and one in each of Gdynia, Łańcut, Łódź, Katowice, Kielce, Kraków, Szczecin, and Wrocław). The COVID-19 and influenza infections in the studied patients were confirmed based on the positive result of the polymerase chain reaction assay or rapid antigen test [19]. All consecutive adult patients hospitalized specifically due to laboratory-confirmed COVID-19 or influenza in participating infectious disease units were included in the analysis, with no exclusions. Patients hospitalized for non-infectious conditions or for chronic diseases without acute viral infection were not included, as such patients are not routinely admitted to infectious disease units and therefore did not constitute a feasible control group within the study design.

The research was a part of the FluTer project run under the auspices of the Polish Association of Epidemiologists and Infectiologists. The statistical comparison of COVID-19 and influenza groups was conducted with the Mann–Whitney and Fisher’s exact tests. Independent predictors of in-hospital death were identified using multivariable logistic regression following univariate analysis of characteristics of survivors and deceased individuals. A *p*-value below 0.05 was considered statistically significant. The study was approved by the Bioethics Committee of the Jan Kochanowski University in Kielce (resolution no. 16/2025, approved: 19 March 2025). Written consent was waived due to the retrospective nature of the research and the preserved anonymity of patients.

## 3. Results

**Figure 1:**
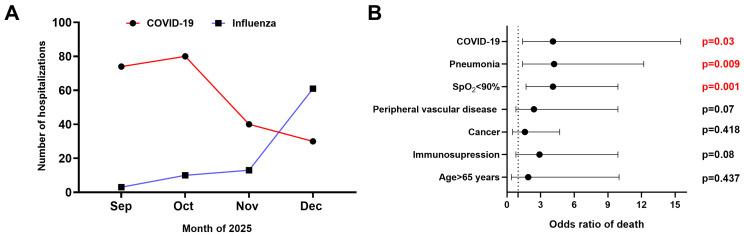
(**A**) The hospitalization burden of COVID-19 and influenza in the participating infectious disease units in the studied period. (**B**) Multivariable analysis of predictors of death in a hospitalized cohort of patients (*n* = 314).

The analysis comprised 314 adult patients hospitalized between September and December 2025 in participating clinical units, including 227 patients admitted with COVID-19 and 87 patients hospitalized with influenza. As shown in Figure 1A, the greatest burden of COVID-19 hospitalizations occurred in September and October 2025, while a sharp increase in influenza-related hospitalizations was seen in December 2025. The patients’ demographic and clinical characteristics are presented in Table 1. The general profiles of both groups were similar, except that patients with COVID-19 were older and more frequently had cancer. Moreover, hospitalization length, baseline oxygen saturation, and CRP levels did not differ between the groups. In contrast, the mortality rate among COVID-19 patients was approximately twofold higher than among those hospitalized with influenza.

Analysis of the entire cohort showed that, compared with survivors, patients who died were more frequently over 65 years of age (92% vs. 76%, *p* = 0.02) and more often had immune deficiency (27% vs. 13%, *p* = 0.03), peripheral vascular disease (54% vs. 31%, *p* = 0.006), cancer (38% vs. 18%, *p* = 0.007), oxygen saturation < 90% (61% vs. 20%, *p* < 0.00001), and developed pneumonia (85% vs. 49%, *p* = 0.00006). Therefore, these variables, along with COVID-19, were included in a multiple logistic regression model to determine their independent effects. As shown in Figure 1B, hospitalization with COVID-19 was independently associated with a fourfold higher risk of death in the entire cohort. Other factors contributing independently to this risk included oxygen saturation below 90% and the development of pneumonia during the course of the disease (Figure 1B). Among patients who died from influenza, none received the latest seasonal vaccine; among patients who died from COVID-19, 84.4% did not receive an updated COVID-19 vaccine.

## 4. Discussion

In this comparative analysis of hospitalized patients during the last quadrimester of 2025, COVID-19 accounted for a substantially higher number of admissions to infectious disease wards than influenza. These findings describe the relative burden of hospitalizations observed within participating infectious disease units rather than population-level incidence or nationwide hospitalization rates. Despite broadly similar clinical profiles between the two groups, COVID-19 patients were significantly older and more frequently affected by cancer, factors known to contribute to severe disease and poor outcomes. This is in line with other comparisons of COVID-19 and influenza patients [16]. Importantly, hospitalization length, baseline oxygen saturation, frequency of pneumonia, and inflammatory markers did not differ significantly, suggesting that differences in outcomes were not merely driven by disease severity at admission.

With the emergence of the Omicron SARS-CoV-2 lineage, numerous studies reported a significant decrease in COVID-19 severity [4,20], a phenomenon linked to distinctive biological changes in the virus [21,22] and to broad immunization through natural infections and global vaccination efforts [23]. This said, our study shows that, at least in the last year’s quadrimester, COVID-19 continues to exert a significant burden, causing hospitalizations, with a mortality rate of 14% among hospitalized patients. Despite this inherent reduction in virulence, the sheer transmissibility of Omicron descendants and the waning of population immunity can still drive significant hospitalization rates, especially among vulnerable groups, as seen in our hospitalized patient cohort.

An important finding is the approximately twofold higher mortality among hospitalized COVID-19 patients compared with those admitted for influenza, with COVID-19 being independently associated with a fourfold increased risk of death, even after adjusting for age, immune deficiency, peripheral vascular disease, hypoxemia, and pneumonia. This association should be interpreted cautiously and does not imply an intrinsic difference in virulence between SARS-CoV-2 and influenza viruses. Rather, it reflects outcomes observed among hospitalized patients within a specific healthcare setting and time period. Potential influences of unmeasured confounders, including frailty, functional status, differences in treatment approaches, and admission thresholds, cannot be excluded despite multivariable adjustment. In addition, the smaller size of the influenza cohort and the low absolute number of influenza-related deaths introduce statistical uncertainty, which may affect the precision and stability of relative risk estimates.

Nevertheless, the findings of the present study highlight COVID-19 as a stronger determinant of in-hospital mortality than influenza during the study period, reinforcing its continued clinical relevance beyond the acute pandemic phase. However, one should note that although COVID-19 remained independently associated with in-hospital death after multivariable adjustment, this association should be interpreted as non-causal and potentially influenced by residual confounding.

These findings have implications when interpreted alongside recent influenza epidemiology. Data from 2024 demonstrated an unusually early and intense influenza season, with December accounting for a disproportionate share of hospitalizations and deaths. Although influenza is typically expected to peak in January or later, this earlier rise suggests that the overlap between COVID-19 and influenza is widening, particularly at the end of the year. In this context, the dominance of COVID-19 hospitalizations observed in September–November 2025 is especially concerning, as it adds to the already increasing influenza burden.

Our finding aligns with the broader epidemiological context of the 2025–2026 respiratory season in the Northern Hemisphere. While a severe influenza A(H3N2) season was underway, marked by an unusually early start, COVID-19 activity remained a significant contributor to hospital burden throughout the period. These observations are intended to contextualize, rather than directly explain, the hospitalization patterns observed in our cohort. The emergence of a new influenza A(H3N2) subclade, designated as subclade K, which became globally predominant and showed antigenic differences from the vaccine strain, contributed to the intensity of the influenza season [24,25].

From a public-health perspective, the higher frequency of COVID-19 hospitalizations and their stronger association with mortality suggest reconsideration of the timing of COVID-19 vaccination updates. In 2024 and 2025, these updated formulations have been available in Poland since October-November. Earlier availability, potentially before September, may be beneficial, particularly for older adults and patients with cancer or vascular disease. However, at this point, these considerations should be viewed as hypothesis-generating rather than prescriptive. They are derived from hospital-based observations and not from direct evaluation of vaccine effectiveness or population-level outcomes.

The coexistence of two high-impact viral pathogens during the studied period, and especially during December, underscores the need for integrated surveillance (including wastewater surveillance), vaccination, and preparedness strategies rather than pathogen-specific approaches [26,27,28,29]. However, it needs to be stressed that the findings pertain to hospitalized patients and should not be extrapolated to population-level lethality or infection fatality risk.

The study has two important strengths. First, it directly compares COVID-19 and influenza hospitalizations within the same clinical unit and time period, minimizing bias related to differences in admission criteria, clinical management, and healthcare access. Second, the focus on the last quadrimester of the year captures a critical, often underexamined period when respiratory viral infections begin to overlap, and healthcare systems face increasing pressure. At the same time, study limitations should also be acknowledged. First, the relatively smaller number of influenza patients compared with COVID-19 cases may have reduced statistical power to detect more subtle differences between the groups. This imbalance also limits the robustness of mortality comparisons, as estimates derived from a small number of events in the influenza group are inherently less precise and should be interpreted with caution. Second, as an observational study, causality cannot be inferred, and residual confounding cannot be fully excluded despite multivariable adjustment, particularly given the older age and higher prevalence of malignancy among COVID-19 patients. Third, although vaccination status was recorded, the study did not evaluate the relationship between time since vaccination and antibody titers, as serological data were not routinely available in this retrospective hospital-based cohort. Consequently, vaccine-related effects could not be analyzed in relation to immune response or antibody waning, and vaccination status was assessed only as a categorical variable.

## 5. Conclusions

During the last quadrimester of 2025, COVID-19 caused more hospitalizations in Poland within participating infectious disease units and was associated with a significantly higher risk of death than influenza among hospitalized patients. Though other studies have evidenced that COVID-19 severity decreased, it still causes a substantial healthcare burden. Moreover, the findings suggest the potential value of exploring earlier COVID-19 vaccination strategies in future prospective studies, and highlight the need for improved healthcare preparedness at year-end, when COVID-19 and influenza increasingly overlap.

## Figures and Tables

**Table 1:** Comparison of demographic and clinical parameters of patients hospitalized with COVID-19 and influenza in September–December 2025. The statistically significant differences (*p* < 0.05 in the Mann–Whitney U test or Fisher’s exact test) are highlighted in bold.

Parameter	COVID-19 (*n* = 227)	Influenza (*n* = 86)	*p*-Value
Age, years, mean ± SD	74.8 ± 15.5	67.0 ± 20.2	**0.003**
Age > 65 years, % (*n*)	82 (187)	66 (57)	**0.001**
Sex, male/female, % (*n*)	47 (106)/53 (121)	47 (41)/53 (46)	0.539
BMI, kg/m^2^, mean ± SD	26.5 ± 5.5	26.7 ± 6.2	0.821
Obesity (BMI ≥ 30 kg/m^2^), % (*n*)	23 (29)	27 (16)	0.345
Ischemic heart disease, % (*n*)	37 (84)	32 (28)	0.254
Peripheral vascular disease, % (*n*)	34 (78)	33 (29)	0.487
Arterial hypertension, % (*n*)	68 (155)	66 (57)	0.367
Diabetes, % (*n*)	33 (74)	30 (26)	0.375
Cancer, % (*n*)	23 (53)	13 (11)	**0.03**
Chronic kidney disease, % (*n*)	17 (39)	17 (15)	0.555
Asthma, % (*n*)	4 (8)	6 (5)	0.275
Chronic obstructive pulmonary disease, % (*n*)	10 (23)	11 (13)	0.324
Immune deficiency, % (*n*)	17 (38)	10 (9)	0.104
Vaccination with the latest vaccine	14 (33)	8 (7)	0.134
Hospitalization length, days, mean ± SD	8.1 ± 6.4	8.2 ± 8.5	0.665
Baseline SpO_2_, %, mean ± SD	74.7 ± 36.3	68.4 ± 41.6	0.940
Baseline SpO_2_ < 90%, % (***n***)	24.4 (49)	23.4 (19)	0.500
Baseline CRP, mg/L, mean ± SD	69.4 ± 73.2	63.2 ± 55.1	0.509
Pneumonia, % (*n*)	53.2 (107)	53.1 (43)	0.543
Death, % (*n*)	14 (31)	6 (5)	**0.03**

BMI—Body Mass Index; CRP—C-reactive levels; SD—standard deviation; SpO_2_—oxygen saturation.

## Data Availability

The data that support the findings of this study are available from the corresponding author upon reasonable request.
